# Coprecipitation of Class II NSAIDs with Polymers for Oral Delivery

**DOI:** 10.3390/polym15040954

**Published:** 2023-02-15

**Authors:** Iolanda De Marco

**Affiliations:** 1 Department of Industrial Engineering, University of Salerno, Via Giovanni Paolo II, 132, 84084 Fisciano, Italy; idemarco@unisa.it; 2 Research Centre for Biomaterials BIONAM, University of Salerno, Via Giovanni Paolo II, 132, 84084 Fisciano, Italy

**Keywords:** coprecipitated particles, ibuprofen, ketoprofen, diclofenac sodium, in vitro and in vivo studies

## Abstract

Non-steroidal anti-inflammatory drugs (NSAIDs) are frequently administered orally with modified-release formulations. The attainment of modified-release drugs is commonly achieved through the coprecipitation of the active principle with a biodegradable polymeric carrier in the form of micro or nanoparticles. In this review, some coprecipitation studies of three highly prescribed NSAIDs (in particular, ibuprofen, ketoprofen, and diclofenac sodium) have been analyzed. The techniques employed to micronize the powder, the polymers used, and the main results have been classified according to the type of release required in different categories, such as delayed, immediate, prolonged, sustained, and targeted release formulations. Indeed, depending on the pathology to be treated, it is possible to achieve specific therapeutic objectives, ensuring that the drug is released at a higher or lower dissolution rate (if compared to conventional drugs) and/or at a different time and/or in a specific site of action.

## 1. Introduction

Pain and swelling are commonly mitigated using anti-inflammatory drugs, divided into two broad categories: corticosteroids and non-steroidal anti-inflammatory drugs (NSAIDs). Corticosteroids are steroid hormones produced in the adrenal cortex of vertebrates or synthetically. They are effective in reducing inflammation, particularly in the treatment of ocular problems [1], pulmonary diseases [2], and hepatitis [3]; considering the side effects linked to their administrations, it is recommended to use them in low doses and for short periods [4]. For these reasons, in the case of not particularly severe inflammations, patients prefer to use NSAIDs which can be purchased, in most cases, without a prescription. The mechanism of action of NSAIDs is based on the inhibition of cyclooxygenase enzymes (COX); pain and inflammation are reduced by restraining the formation of prostaglandins [5]. It is well known that COX exists as three isoforms: cyclooxygenase-1 (COX-1) is constitutive, typically expressed in cells and present, for example, in the endothelium, stomach and kidney; cyclooxygenase-2 (COX-2) is a form not always functional inside cells, which is induced by proinflammatory cytokines and endotoxin; cyclooxygenase-3 (COX-3) is believed to be centrally located, although many of its characteristics and functions remain currently ununderstood [6]. Considerations concerning the latter form will, therefore, be excluded from this review paper.

**Figure 1:**
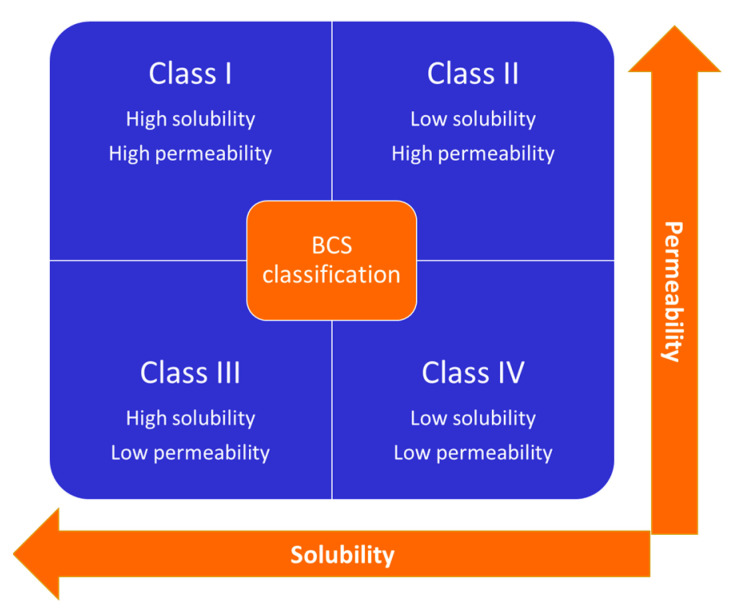
Biopharmaceutical Classification System (BCS) for drugs.

Some NSAIDs are COX-1 selective, some are non-selective, and others are COX-2 selective. NSAIDs are extensively used for their analgesic, antipyretic and anti-inflammatory properties and treat many inflammatory conditions or pain, such as headaches, toothache, soft tissue injuries, rheumatoid arthritis, osteoarthritis, etc. [7,8,9]. Recently NSAIDs, in particular ibuprofen, have been widely used in the home management of non-serious cases of COVID-19 [10,11], in therapies involving the use of NSAIDs and antioxidant compounds or vitamin complexes [12,13,14]. One of the main limitations of the use of NSAIDs is linked to their poor water solubility. Indeed, according to the Biopharmaceutical Classification System (BCS), drugs are classified into four classes based on their solubility and permeability, as represented in Figure 1. Among them, class II drugs are characterized by slow solubilization, high permeation, and low bioavailability. By optimizing the formulation, the absorption of BCS class II drugs can be significantly improved; traditional and innovative methods have been proposed to enhance the bioavailability of these drugs [15]. Among them, the coprecipitation of the active principal ingredient (API) with a suitable polymer has been frequently used to enhance the dissolution rate and, therefore, the bioavailability of the drug [16,17,18].

Given the vastness of the topic and considering that many NSAIDs are characterized by low solubility and high permeability, this review focuses on some BCS class II NSAIDs frequently prescribed or used by patients. The purpose of this review is linked to the need for an analysis that allows the identification of possible formulations not still present on the market, which could complement the already existing ones with reduced side effects. The formulation of new products could also expand the offer by addressing the problem that has recently arisen regarding the shortage of some drugs.

## 2. NSAIDs + Polymer Coprecipitation

Different techniques have been employed to coprecipitate an NSAID with a polymer carrier. Depending on the application, it is essential to correctly choose the carrier to obtain the drug’s release at the desired speed and/or to a specific site of action. Indeed, the release of the active principle strongly depends on the carrier’s affinity with water: hydrophilic polymers, such as PVP, can be used to enhance the drug dissolution rate; hydrophobic polymers, such as zein, can be used when a prolonged release is desired; pH-sensitive polymers, such as Eudragit, can be used for a targeted drug release [19,20,21]. This review is organized into subsections dedicated to three widely used NSAIDs; the published papers are classified as follows: (a) ibuprofen; (b) ketoprofen; (c) diclofenac sodium. In particular, ibuprofen is a non-selective drug towards COX-1 and COX-2; ketoprofen is COX-1 selective, whereas diclofenac sodium is COX-2 selective. Furthermore, attention has been focused on the type of formulations in terms of drug release for these three active ingredients. It is known that the release of a drug can be controlled by acting on the:

- time of release (for example, delayed, repeated, or pulsatile release drugs);
- dissolution rate (reduction or increase of the dissolution rate, obtaining prolonged or immediate release formulations);
- place (release in specific regions, e.g., gastrointestinal tract).

In the literature analysis, a classification was made into delayed, fast, prolonged (or sustained), controlled, and targeted release formulations. ***Delayed release*** occurs when the drug is released after a latency period. Included in this group are gastro-resistant tablets, which allow the passage of the drug through the stomach, where gastric juices could usually destroy the polymer carrier and where the drug release can cause severe damage. ***Immediate-release*** dosage is a mechanism that delivers a drug without delaying or prolonging dissolution. ***Prolonged-release*** pharmaceutical forms are preparations that slowly release the active principle over time, extending the duration of its effectiveness compared with a conventional pharmaceutical form. ***Sustained-release*** pharmaceutical forms are preparations that, like the previous ones, slowly release the drug over time, but compared to them, they release the drug at a predetermined rate maintaining constant API concentration for a specific time. ***Targeted release*** dosage is a mechanism that delivers the drug to a specific target in the body.

### 2.1. Ibuprofen

Ibuprofen first came to market about 50 years ago. It has a balanced safety profile due to its non-selectivity between COX-1 and COX-2 [22]. In any case, as well as other NSAIDs, prolonged use of this active ingredient can damage the upper gastrointestinal tract [23]. For this reason, it has been frequently coupled with a lipidic or polymeric carrier. In Table 1, the attempts to process ibuprofen in the presence of a carrier are listed, together with the technique used to process the NSAID, the morphology obtained, and the main results.

**Figure 2:**
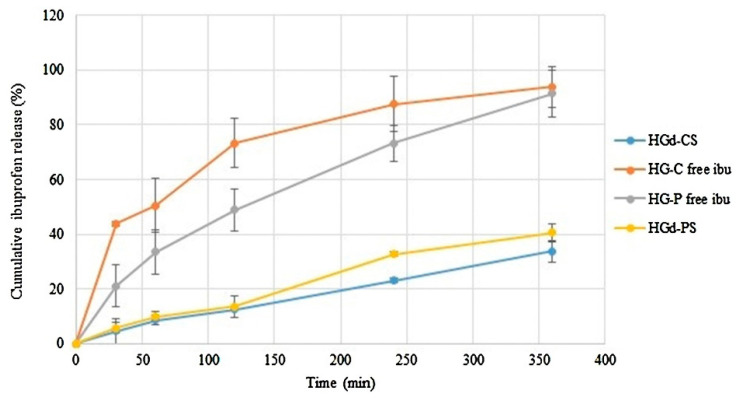
In vitro release profile of ibuprofen from hydrogels with Carbopol^®^ 980 (HGd-CS), hydrogels with polycarbophil (HGd-PS), Carbopol^®^ 980 gel with free ibuprofen (HG-C free ibu) and polycarbophil gel with free ibuprofen (HG-P free ibu). Reprinted with permission from [29]. Copyright© 2017 Elsevier.

As it is possible to observe from Table 1, in many cases, ibuprofen is contained in lipid nanoparticles, which can be distinguished into two categories: solid lipid nanoparticles (SLN), constituted of a solid lipid matrix, and nanostructured lipid carriers (NLC), composed by a blend of a solid lipid and a liquid lipid. The disadvantage of the latter is that, upon administration, they are rapidly removed from the salivary liquid, as they do not remain in contact with the oral mucosa for a sufficient time. To avoid this drawback, they can be incorporated into mucoadhesive preparations, such as the hydrogels proposed by Marques et al. [29]. As shown in Figure 2, if properly designed, mucoadhesive hydrogels allow a sustained release of the active ingredient; gels with free ibuprofen (HG-C free ibu and HG-P free ibu) released the drug faster in comparison with the gels containing NLC.

Different carriers, such as gelatin, chitosan, soy protein, and some others, have been used in the literature to protect ibuprofen and tune its release.

The authors have often stressed the importance of the process yield and encapsulation efficiency to avoid wasting active ingredients. The chosen polymers generally favored the achievement of sustained releases of the active principle [29,31,32,33,34] to reduce the dosing frequency and the adverse gastrointestinal reactions induced by ibuprofen. In some cases, in vitro release kinetics were evaluated in simulated gastrointestinal conditions (pH 1.2 and 6.8), and pH-sensitive release patterns were observed.

**Figure 3:**
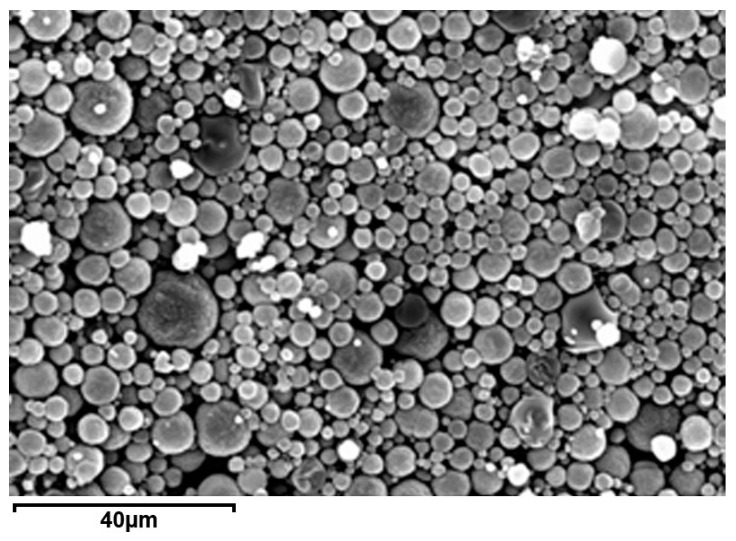
Scanning electron images of chitosan-ibuprofen microparticles obtained by spray drying. Reprinted with permission from [31]. Copyright© 2012 Elsevier.

The different techniques used by the authors allow for obtaining particles with different dimensions and distributions. For example, spherical microparticles of quite regular sizes are obtained through spray drying, as shown in the exemplificative image in Figure 3.

### 2.2. Ketoprofen

Ketoprofen is an NSAID with analgesic, anti-inflammatory, and antipyretic properties synthesized in 1968. It is indicated to relieve mild-to-moderate pain conditions such as dental pain, dysmenorrhea, post-operative pain, and chronic problems such as osteoarthritis and rheumatoid arthritis [37,38]. Adverse events induced by ketoprofen use are headache, cardiovascular reactions, dermatological issues, gastric and duodenal irritation, ulceration, and bleeding [39,40]. To reduce the side effects and suitably modulate the release of the drug, this is associated with a suitable carrier to form a composite system. Similar to what has already been observed for ibuprofen, different techniques and carriers have been used to process this active ingredient. The main results are summarized in Table 2.

Depending on the application, different polymers have been used: to obtain an immediate or rapid effect (for example, in the case of headaches), polymers that speed up the release of the API have been chosen [43,48], to treat inflammatory diseases influenced by circadian rhythms, the aim of targeting early morning symptoms other polymers have been selected [41], or to obtain the release in specific areas of the body, polymers such as eudragits which are pH-dependent have been utilized [48]. In other cases, coprecipitated particles have been obtained, but no dissolution tests were performed. For example, Widyastama and Kumiati [61] optimized the sonication time and surfactant concentration to obtain ketoprofen/chitosan/alginate nanoparticles.

In some cases, coupling a polymer with an inorganic material has given rise to the obtainment of multifunctional products. For example, Attia et al. [46] used an in situ oxidative chemical polymerization of pyrrole coupled with a reduction of ferric chloride in the presence of ketoprofen (with or without a surfactant). The final product is a multifunctional system, which could act as a nanocarrier for drug molecules and a contrasting agent.

**Figure 4:**
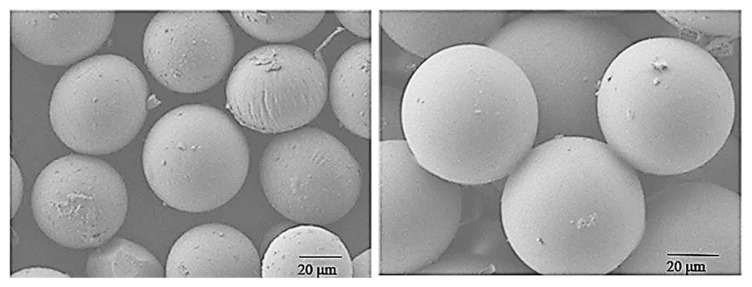
Scanning electron images of ketoprofen-microRNA-PLGA (**left**) and ketoprofen-PLGA (**right**) microparticles. Reprinted with permission from [56]. Copyright© 2018 Elsevier.

Ketoprofen was also coupled with microRNAs, post-transcriptional regulators of gene expression, which are small endogenous non-coding RNAs. Their presence can induce the improvement of the therapeutic effect of ketoprofen when rheumatoid arthritis is treated. Indeed, Yu et al. [56] performed in vivo pharmacodynamics experiments using ketoprofen co-loaded with microRNA-124 into PLGA microspheres (which exemplificative images are reported in Figure 4), observing that ketoprofen could significantly reduce inflammation of the joints and microRNA-124 could reduce bone damage. In addition, they had remarkably advanced activity over the delivery of either microRNA-124 or ketoprofen in suppressing adjuvant-induced arthritis in rats.

In some cases, drugs derived from ketoprofen have been used, such as its lysinated form. This drug can be used for lung inflammation that often occurs in patients with cystic fibrosis. Stigliani et al. used co-spray drying by precipitating ketoprofen lysinate with leucine to obtain microparticles that can reach the deepest airways [62].

**Figure 5:**
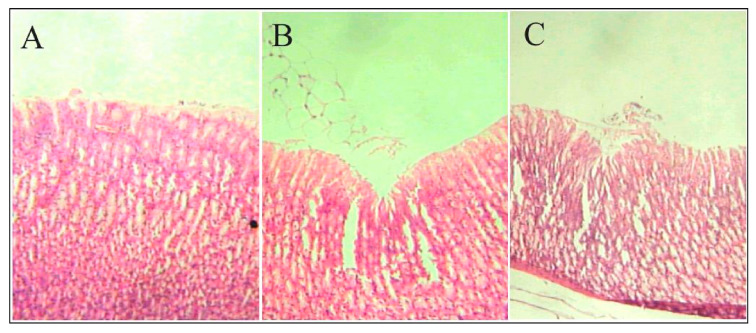
Binocular light microscope images of stomach histopathology of control rats (**A**), rats treated with pristine ketoprofen (**B**) and rats treated with ketoprofen contained in pH-sensitive polyspheres (**C**). Reprinted with permission from [60]. Copyright© 2019 Elsevier.

A further step with respect to in vitro dissolution tests was made by some authors who also carried out in vivo tests [41,56,60]. In some cases, the powders obtained have also been tested on animals. For example, Boppana et al. [60] have prepared pH-sensitive interpenetrated network polyspheres to achieve intestinal targeted delivery of ketoprofen, avoiding API side effects such as ulcer formation, erosion of gastric mucosa, and hemorrhages. The authors conducted tests on rats, administering ketoprofen as it is and ketoprofen contained in the polyspheres. Stomach histopathological studies had shown ulcers (1.97 mm in size), hemorrhages, prominent mucosal erosion with congestion, edema, and perforations when ketoprofen alone was administered; on the contrary, in rats administered with pH-sensitive polyspheres, small ulcers of about 0.11 mm without perforation, congestion, hemorrhages, and necrosis were noticed. Pictures obtained through a binocular light microscope are reported in Figure 5. In particular, in Figure 5A, the stomach of control rats with signs of ulcer/hemorrhages is reported. In contrast, the analyses of the guts of rats treated with pristine ketoprofen or ketoprofen contained in pH-sensitive polyspheres are reported in Figure 5B,C, respectively.

### 2.3. Diclofenac Sodium

Diclofenac was patented in 1965 and came into medical use in 1988. It treats pain and inflammatory diseases, especially arthritis, rheumatoid arthritis, osteoarthritis, dental pain, menstrual pain, and endometriosis. An additional indication is the treatment of acute migraines and moderate postoperative or post-traumatic pain. In some cases, it is administered locally through transdermal delivery [63,64,65] or through in situ injections [66,67], but the most common method of administration is the oral one. The side effects that occur following its oral intake are familiar to those of other NSAIDs. Therefore, it is generally administered in the form of micrometric or nanometric particles after being coupled to a carrier, which modulates the release of the API limiting damage to the stomach. Table 3 summarizes the main literature results regarding processing diclofenac sodium with various techniques.

As already observed in the previous sections, what is immediately noticeable when looking at Table 3 is the variety of polymeric carriers used to process the active ingredient (specifically, diclofenac sodium) to control temporal or distributional drug delivery. The category of polymers that appears to be most used is that of eudragit, which has the particularity of being pH-dependent, in many cases favoring the dissolution of the active ingredient in the intestinal tract. It also seems interesting to use poly electrolyte complexes, constituted by combining two polymers, of which one polymer is positively charged, and another is negatively charged. For example, Sandeep et al. [79] combined cationic guar gum, which has a net positive charge because of its trimethyl ammonium groups, with xanthan gum, with polyanionic properties due to carboxylic groups. These two polymers, despite their biodegradable character, cannot be used alone in the attainment of controlled-release formulations as both possess highly acidic or alkaline pH caused by the presence of anionic or cationic groups. When they react together, a novel poly electrolyte complex can be prepared to release the API in a controlled manner.

It can also be noted that depending on the techniques and operating conditions used, both nanoparticles and microparticles are obtained. Considering that the powders obtained are generally administered in the form of tablets or capsules, no particular advantage is observed in going down to very small sizes. Microparticles, therefore, could have the advantage of being easier to handle than nanoparticles. This allows easier recovery of the micronized material obtained.

## 3. Conclusions

This review focused on the coprecipitation of three NSAIDs with proper polymeric carriers to obtain controlled-release drugs. Various techniques and a wide range of polymers have been used to obtain powders that can be used for different purposes. In the case of ibuprofen and diclofenac sodium, coprecipitated particles were mainly obtained for the sustained or prolonged release of the active ingredient using chitosan and its derivatives, starch, ethyl cellulose, soy proteins, or zein as carriers. Ketoprofen, on the other hand, has been formulated for different purposes. In some cases, it has been coprecipitated with PVP (alone or combined with other polymers) or cyclodextrins to obtain rapid-release formulations; in other cases, delayed-release (with alginate and its derivatives) or sustained-release (using chitosan or PMMA) formulations were prepared. For all the APIs, it was also possible to note a wide use of pH-dependent polymers, such as eudragit, which avoid the dissolution of the active principle inside the stomach, allowing its release at intestinal pH. Some studies have stopped at the in vitro analysis of the dissolution of the active ingredients, while others have gone as far as studying the release with cells in vivo and on animals (mice or rabbits).

## Figures and Tables

**Table 1:** Ibuprofen processing. HPH = high pressure homogenization; HSH = high speed homogenization; LNC = lipid nanocapsules; LS = lipospheres; MC = microcapsules; m.d. = mean diameter; MH = mucoadhesive hydrogels; MPs = microparticles; MT = mucoadhesive tablets; NLC = nanostructured lipid carriers; NPs = nanoparticles; poly(mPEGMA-*co*-MAA) = methoxy poly(ethylene glycol) methacrylate-*co*-poly(methylacrylic acid) copolymer; SGF = simulated gastric fluid; SIF = simulated intestinal fluid; SSS = simulated saliva solution.

Morphology	Technique	Carrier	Main Results	Reference
***Delayed release formulations***				
MPs	Spray-drying	Succinylated soy protein	m.d. = 4 ÷ 8 μm; encapsulation efficiency up to 95%; dependence of the release from the pH, demonstrating that a delayed release of the API in the gastrointestinal tract can be obtained	[24]
***Immediate release formulations***				
MC	Spray drying	Gelatin	m.d. = 6.34 ± 0.57 μm; amount of NSAID dissolved from gelatin microcapsule in SGF increased fivefold compared to ibuprofen powder; enhanced oral bioavailability demonstrated through in vivo experiments on rats	[25]
***Prolonged release formulations***				
LNC	Phase inversion	Triglyceride	m.d. = 45 ÷ 60 nm; in vitro controlled drug release in SIF within 24 h useful for intravenous administration; in vivo experiments on rats demonstrated a prolonged efficiency of LNC after oral administration	[26]
LS	Hot emulsification	Phospholipon^®^ 90H and beeswax	m.d. = 137 ÷ 178 μm; entrapment efficiency in the range 89.4 ÷ 97.9%; analgesic and anti-inflammatory activities achieved with prolonged plasma concentration demonstrated through in vivo experiments on rats	[27]
NPs	Semicontinuous heterophase polymerization	Poly(ethyl cyanoacrylate)	m.d. = 10 ÷ 58 nm; particles with such low diameters would remain for long periods in the blood stream because they would be able to avoid renal removal	[28]
***Sustained release formulations***				
MH with NLC	HPH or HSH	Carbopol^®^ 980 and polycarbophil	NLC dispersions in the nanometric size range with low polydispersity index values and efficient ability for the entrapment of the API; in vitro release studies in SSS revealed a sustained release of the drug	[29]
MPs	Spray-drying	Chitosan/xanthan gum	In vitro release tests in SGF and SIF revealed a constant drug release rate during 12 h, with approximately 30% of ibuprofen released, but with a tendency for further release over a more extended period	[30]
MT	Spray drying	Chitosan and its half-acetylated derivative	Drug loading of 41% (close to the theoretical loading) in the case of chitosan; dissolution tests performed at pH 2.0 (polymers dissolve, API poorly soluble) and pH 7.0 (API dissolves quickly, chitosan insoluble); sustained release of the API	[31]
NPs	Lyophilization	Chitosan-modified poly(mPEGMA-co-MAA)	In vitro release profiles in SGF and SIF show that the API is released in more than 24 h; the dosing frequency can be reduced. In vivo experiments on rats showed a good antifebrile effect	[32]
NPs	Homogenization of a bulk cubic phase gel into a cubic dispersion	Phytantriol/poloxamer 407	m.d. = 238 nm; encapsulation efficiency higher than 85%; sustained release demonstrated by in vitro tests in SGF and SIF conditions; in vivo pharmacokinetic studies in beagle dogs showed improved absorption of ibuprofen compared to the pure API’s one	[33]
NPs	Nanoprecipitation	Starch citrate and phosphate	m.d. = 616 ÷ 933 nm; sustained delivery up to 24 h demonstrated by in vitro tests performed at pH 6.8	[34]
***Targeted release formulations***				
MPs	Emulsification-cross-linking	Chitosan	m.d. = 326.70 ± 10.43 μm; yield up to 99.2%; in vitro profiles at SGF and SIF show a targeted and controlled drug release with a biphasic pattern	[35]
MPs	Spray-drying	Soy protein isolate and acylated soy protein	Yields in the range 70 ÷ 87% and encapsulation efficiencies higher than 80%; pH-sensitive release patterns evaluated through in vitro release tests in SGF and SIF	[36]

**Table 2:** Ketoprofen processing. CDR = cumulative drug release; EE = encapsulation efficiency; EPI-βCd = β-cyclodextrin-epichlorohydrin polymer; EPI-CMβCd = carboxymethylathed-β-cyclodextrin-epichlorohydrin polymer; HPMC = hydroxypropyl methylcellulose; MC = microcapsules; m.d. = mean diameter; MPs = microparticles; NPs = nanoparticles; PAAm-g-LBG = polyacrylamide-g-locust bean gum; PHB = Poly(3-hydroxybutyrate); PLGA = Poly(lactic-co-glycolic acid); PMMA = poly(methyl methacrylate); PVP = polyvinylpyrrolidone; PVPVA = poly(vinylpyrrolidone-co-vinyl acetate); SAS = supercritical antisolvent; SD = solid dispersion; SGF = simulated gastric fluid; SIF = simulated intestinal fluid; SMA = poly(styrene-co-maleic acid) copolymer.

Morphology	Technique	Carrier	Main Results	Reference
***Delayed release formulations***				
Beads	Prilling/ionotropic gelation	Zn-alginate	Delayed release of the drug in SIF; able to control early morning clinical symptoms following the circadian rhythm of the proinflammatory mediators	[41]
MPs	Ionic gelation	Sericin-alginate crosslinked with proanthocyanidin	m.d = 1.36 ÷ 1.47 μm; EE = 91.1%; gastroresistant particles with a delayed release in 6 h (in SIF)	[42]
***Immediate release formulations***				
MPs	SAS process	PVP	m.d. = 2.4 ÷ 3.8 μm; increase in the drug dissolution rate (evaluated in SGF) of about 4.2 times with respect to the unprocessed API	[43]
MPs	Spray-drying	Eudragit E + PVP, PVPVA or HPMC	m.d. = 6.9 ± 2.0 μm; the API release was much faster compared to a commercially available product and the dissolution of the unprocessed drug; verified the stability of the formulations during storage at room-temperature conditions in open vials	[44]
MPs	Electrospraying and spray-drying	PVP and PVPVA	Comparing electrospraying spray-drying, smaller particles were obtained using PVP (m.d. = 0.79 ± 0.02 μm instead of 1.11 ± 0.08 μm) and bigger particles were obtained using PVPVA (m.d. = 1.12 ± 0.05 μm instead of 0.95 ± 0.06 μm); high loadings (up to 97.4%); fast drug release in SGF	[45]
NPs	Chemical oxidative polymerization	Hybrid iron oxide/polypyrrole	m.d. well below 50 nm; EE equal to 98%; complete drug release after 3 h; multifunctional final product	[46]
NPs	Co-grinding	EPI-βCd or EPI-CMβCd	m.d. = 300 ÷ 436 nm; EE up to 77%; marked increase in the drug release rate using EPI-βCd as the carrier	[47]
NPs	Co-lyophilization	EPI-βCd or EPI-CMβCd	m.d. = 298÷494 nm; EE up to 58.4%	[47]
SD	Solvent evaporation	Poloxamer 188	Drug content in the range 83 ÷ 92%; dissolution rate after 90 min clearly improved (CDR = 92%) in comparison with the unprocessed API (CDR = 28%) and the physical mixture (CDR = 66%)	[48]
SD	Melting/fusion	Eudragit S 100	Drug content in the range 85 ÷ 91%; pH-dependent dissolution rate after 90 min clearly improved (CDR = 96%) in comparison with the unprocessed API (CDR = 28%) and the physical mixture (CDR = 69%)	[48]
SD	Kneading and solvent evaporation	PVP K30	Drug entrapped within the carrier matrix; increased dissolution rate with respect to that of the pure drug	[49]
SD	Kneading and melting	Mannitol	Drug adsorbed as fine particles on the surface of the carrier; increased dissolution rate with respect to that of the pure drug	[49]
***Prolonged release formulations***				
Beads	Prilling/ionotropic gelation	Pectin + Eudragit S100	EE up to 87%; prolonged release in SIF until 5 h; formulation potentially effective for the chronotherapy of early morning pathologies	[50]
MPs	Emulsification-solvent evaporation	Ethylcellulose and Eudragit RL 100	m.d. = 149.2 ± 1.25 μm; EE up to 90%; prolonged drug release for 8 h (91.25% of the API)	[51]
Particles	Ionic gelation	Sericin/alginate blend	Improved thermal stability of the drug after incorporation into the blend; comparison with a commercially available drug (Enteric Profenid^®^) showed a slower release and higher drug release value, demonstrating superior results without the use of excipients	[52]
***Sustained release formulations***				
MC	Solvent evaporation	Eudragit RS + aluminum tristearate	Spherical MC with a diameter smaller with an increasing amount of aluminum tristearate; sustained release of ketoprofen	[53]
MPs	Emulsification-solvent evaporation	PHB/chitosan	m.d = 31.33 ÷ 40.34 μm; release with a pronounced burst effect in absence of chitosan; sustained release of the API using chitosan	[54]
MPs	Solvent evaporation	Eudragit RSPO	m.d. = 123 ÷ 310 μm; EE up to 92%; sustained drug release in correspondence of the optimized operating conditions	[55]
MPs	Multiple emulsion-solvent evaporation	PLGA	Spherical MPs with a m.d. of 47.37 ± 37.5 μm (in the case of the API) and 53.46 ± 38.4 μm (when the API was co-loaded with microRNA-124; initial burst followed by a sustained release; in vivo demonstration of the reduction of the inflammation due to rheumatoid arthritis	[56]
NPs	Nanoprecipitation + spray drying	PMMA	Highly monomodal particle in the size range 100 ÷ 210 nm; sustained drug release over 6 h	[57]
Particles	Drying at 40 °C	PVP K30 coated with TiO_2_	Sustained release of the API for 8 h obtained by coating the particles with TiO_2_ (evaluated in SGF conditions)	[58]
***Targeted release formulations***				
NPs	Precipitation	SMA	Spherical particles with m.d. varying from a few tens of nanometers to micrometers; targeted pH-mediated drug delivery	[59]
Particles	Ambient drying	PAAm-g-LBG/sodium alginate	m.d. = 857 ÷ 948 μm; targeted delivery of ketoprofen to small intestine; reduced side effects such as ulcer formation, erosion of gastric mucosa, and hemorrhages, demonstrated through stomach histopathological studies on rats	[60]

**Table 3:** Diclofenac sodium processing. BALB = mouse embryonic fibroblasts; Caco2 = human colon carcinoma cells; EE = encapsulation efficiency; GIT = gastro-intestinal tract; HPβCd = hydroxypropyl-β-cyclodextrin; HPMC = hydroxypropyl methylcellulose; m.d. = mean diameter; LAS = liquid antisolvent; MPEG-PCL = methoxypoly(ethylene glycol)-poly(3-caprolactone); MPs = microparticles; NIPAAm = N-isopropyacrylamide; NPs = nanoparticles; PEG = poly(ethylene glycol); PGA-co-PDL = Poly(glycerol adipate-co-x-pentadecalactone); PGA-co-PDL-PEGme = poly(ethylene glycol methyl ether)-Poly(glycerol adipate-co-x-pentadecalactone); PHBCL = poly(3-hydroxybutyrate-co-ε-caprolactone); PLGA = Poly(lactic-co-glycolic acid); PVA = poly vinyl alcohol; SAS = supercritical antisolvent.

Morphology	Technique	Carrier	Main Results	Reference
			***Delayed release formulations***	
MPs	Spray drying	Ethylcellulose or Eudragit RS30D	m.d. = 16.19 ÷ 35.11 μm using ethyl cellulose and 14.83 ÷ 40.06 μm using Eudragit RS30D; EE = 64.7% using ethyl cellulose and 54.0% using EudragitRS30D; delayed API dissolution rates, sustaining the release for several hours	[68]
MPs	Emulsification/internal gelation	Sodium alginate	m.d. in the range 200 ÷ 630 μm depending on the operating conditions; EE up to 96.3%; in vitro releases performed in SGF and SIF conditions	[69]
MPs	Spray drying	Corn starch + pectin	m.d. in the range 5.8 ÷ 7.3 μm; controlled release of the drug in the lower part of the GIT	[70]
			***Immediate release formulations***	
MPs	Solvent evaporation	Chitosan + Ethylcellulose	m.d. in the range 50 ÷ 70 μm; influence of the core/coat ratio on the in API in vitro release	[71]
			***Prolonged release formulations***	
MPs	SAS	Zein	m.d. in the range 0.31 ÷ 1.31 μm; prolonged drug release at the optimized operating conditions	[72]
MPs	Solvent deposition	PHBCL	m.d. in the range 0.5 ÷ 4.5 μm; absence of relevant toxicity effects on Caco2 and BALB mice	[73]
MPs	Emulsion cross-linking	Xanthan gum + PVA	m.d. in the range 310.25 ÷ 477.10 μm; EE up to 82.94%; prolonged drug release demonstrated through in vivo tests on rabbits	[74]
MPs	Single emulsion and solvent evaporation	Ethylcellulose	m.d. in the range 10 ÷ 200 μm; EE up to 84.9%; prolonged drug release over 12 h	[75]
NPs	Single-emulsion solvent diffusion	Eudragit RS100	m.d. = 103 ÷ 170 nm; controlled release of the API for an extended time	[76]
NPs	Template polymerization and freeze-drying	Chitosan-poly(methacrylic acid)	Uniform size in the range 50 ÷ 100 nm; drug release prolonged over 48 h (promising for modified release systems development)	[77]
			***Sustained release formulations***	
Micelles	Solvent diffusion	MPEG-PCL	Particles in the range 54.1 ÷ 94.4 nm; in vitro sustained release (ocular delivery) and good corneal penetration; in vivo tests on rabbit eyes demonstrated a good bioavailability	[78]
MPs	Drying	Cationic guar gum + xanthan gum	m.d. in the range 294 ÷ 300 μm; EE up to 96.47%; drug release extended beyond 12 h demonstrated by in vitro release studies; in vivo studies on rabbits showed the sustained release of the API	[79]
MPs	Radical copolymerization	NIPAAm and gelatin	m.d. in the range 80 ÷ 130 μm depending on the crosslinker amount in the polymeric networks	[80]
MPs	Single emulsion and spray drying	PEG-PGA-co-PDL-PEG and PGA-co-PDL-PEGme	m.d. up to 3.92 ± 0.12 μm; EE up to 60.88%; initial burst followed by a sustained release till 24 h (useful for lung delivery)	[81]
MPs	LAS	Ethylcellulose	Irregular and aggregated particles in the range 390 ÷ 442 μm; sustained release of the API	[82]
MPs	Spray drying	Sweet potato starch	m.d. in the range 10.3 ÷ 13.1 μm; EE up to 98.2%; sustained release over a period of 6 h	[83]
NPs	Crosslinking precipitation	Hydroxyethyl starch	m.d. = 170 ± 5 nm; EE = 72 ± 3%; sustained drug release for 72 h; the API achieved clinical therapeutic levels in the blood for up to 120 h, with minimal accumulation in critical organs	[84]
NPs	Nanoprecipitation	Eudragit L100	Particles in the range from 87 ± 0.47 nm to 103 ± 0.26 nm; EE up to 99.03%; initial burst effect followed by a sustained release; arthritis was induced in rabbits, which were treated with intraarticular injections of the API, demonstrating a significant reduction in swelling	[85]
NPs	Emulsification and solvent evaporation	Ethylcellulose	m.d. = 226.8 nm; EE up to 49.1%; sustained release of the API	[86]
NPs	Emulsification and evaporation under reduced pressure	Eudragit L100 or Eudragit L100/PLGA	m.d. = 241 ÷ 274 nm; EE up to 62%; initial burst release followed by a slower sustained release; release profiles and EE dependent on the amount of Eudragit in the blend	[87]
			***Targeted release formulations***	
NPs	Emulsification and evaporation	HPβCd and/or EudragitL100	m.d. = 385 ÷ 663 nm; colon-targeted NPs for transmucosal delivery of the API; drug permeation through colonic pig mucosa and Caco2 cell line	[88]
MPs	Spray drying	Mannitol, maltodextrin or HPMC	m.d. in the range 1.5 ÷ 6.4 μm depending on the carrier; pH-dependent drug release to obtain an intestinal drug delivery	[89]

## Data Availability

Not applicable.

## References

[1] Muñoz-Fernández S., Martín-Mola E. (2006). Uveitis. Best Pract. Res. Clin. Rheumatol..

[2] Diette G.B., Dalal A.A., D’souza A.O., Lunacsek O.E., Nagar S.P. (2015). Treatment patterns of chronic obstructive pulmonary disease in employed adults in the United States. Int. J. COPD.

[3] Yeoman A.D., Westbrook R.H., Zen Y., Bernal W., Al-Chalabi T., Wendon J.A., O’Grady J.G., Heneghan M.A. (2014). Prognosis of acute severe autoimmune hepatitis (AS-AIH): The role of corticosteroids in modifying outcome. J. Hepatol..

[4] Del Grossi Moura M., Cruz Lopes L., Silva M.T., Barberato-Filho S., Motta R.H.L., Bergamaschi C.C. (2018). Use of steroid and nonsteroidal anti-inflammatories in the treatment of rheumatoid arthritis: Systematic review protocol. Medicine.

[5] Whittle B.J.R. (2000). COX-1 and COX-2 products in the gut: Therapeutic impact of COX-2 inhibitors. Gut.

[6] Vane J.R., Bakhle Y.S., Botting R.M. (1998). Cyclooxygenases 1 and 2. Annu. Rev. Pharmacol. Toxicol..

[7] Gupta A., Bah M. (2016). NSAIDs in the Treatment of Postoperative Pain. Curr. Pain Headache Rep..

[8] Maniar K.H., Jones I.A., Gopalakrishna R., Vangsness C.T. (2018). Lowering side effects of NSAID usage in osteoarthritis: Recent attempts at minimizing dosage. Expert Opin. Pharmacother..

[9] Franco P., De Marco I. (2020). Supercritical CO_2_ adsorption of non-steroidal anti-inflammatory drugs into biopolymer aerogels. J. CO2 Util..

[10] García N.H., Porta D.J., Alasino R.V., Muñoz S.E., Beltramo D.M. (2020). Ibuprofen, a traditional drug that may impact the course of COVID-19 new effective formulation in nebulizable solution. Med. Hypotheses.

[11] Abu Esba L.C., Alqahtani R.A., Thomas A., Shamas N., Alswaidan L., Mardawi G. (2021). Ibuprofen and NSAID Use in COVID-19 Infected Patients Is Not Associated with Worse Outcomes: A Prospective Cohort Study. Infect. Dis. Ther..

[12] De Marco I. (2022). Production of carrier/antioxidant particles by Supercritical Assisted Atomization as an adjuvant treatment of the COVID-19 pathology. J. Supercrit. Fluids.

[13] Sharifi S., Samani A., Ahmadian E., Eftekhari A., Derakhshankhah H., Jafari S., Mokhtarpour M., Vahed S.Z., Salatin S., Dizaj S.M. (2019). Oral delivery of proteins and peptides by mucoadhesive nanoparticles. Biointerface Res. Appl. Chem..

[14] Chodari L., Dilsiz Aytemir M., Vahedi P., Alipour M., Vahed S.Z., Khatibi S.M.H., Ahmadian E., Ardalan M., Eftekhari A. (2021). Targeting mitochondrial biogenesis with polyphenol compounds. Oxidative Med. Cell. Longev..

[15] Kansara H., Panola R., Mishra A. (2015). Techniques used to enhance bioavailability of bcs class II drugs: A review. Int. J. Drug Dev. Res..

[16] Mammucari R., Dehghani F., Foster N.R. (2006). Dense gas processing of micron-sized drug formulations incorporating hydroxypropylated and methylated beta-cyclodextrin. Pharm. Res..

[17] Vemavarapu C., Mollan M.J., Needham T.E. (2009). Coprecipitation of pharmaceutical actives and their structurally related additives by the RESS process. Powder Technol..

[18] Franco P., De Marco I. (2020). Supercritical Antisolvent Process for Pharmaceutical Applications: A Review. Processes.

[19] Yu D.G., Shen X.X., Branford-White C., White K., Zhu L.M., Annie Bligh S.W. (2009). Oral fast-dissolving drug delivery membranes prepared from electrospun polyvinylpyrrolidone ultrafine fibers. Nanotechnology.

[20] Lai L.F., Guo H.X. (2011). Preparation of new 5-fluorouracil-loaded zein nanoparticles for liver targeting. Int. J. Pharm..

[21] Franco P., De Marco I. (2020). Eudragit: A novel carrier for controlled drug delivery in supercritical antisolvent coprecipitation. Polymers.

[22] Varrassi G., Pergolizzi J.V., Dowling P., Paladini A. (2020). Ibuprofen Safety at the Golden Anniversary: Are all NSAIDs the Same? A Narrative Review. Adv. Ther..

[23] Somasundaram S., Hayllar H., Rafi S., Wrigglesworth J., Macpherson A., Bjarnason I. (1995). The biochemical basis of non-steroidal anti-inflammatory drug-induced damage to the gastrointestinal tract: A review and a hypothesis. Scand. J. Gastroenterol..

[24] Anaya Castro M.A., Alric I., Brouillet F., Peydecastaing J., Fullana S.G., Durrieu V. (2019). Spray-Dried Succinylated Soy Protein Microparticles for Oral Ibuprofen Delivery. AAPS PharmSciTech.

[25] Li D.X., Oh Y.K., Lim S.J., Kim J.O., Yang H.J., Sung J.H., Yong C.S., Choi H.G. (2008). Novel gelatin microcapsule with bioavailability enhancement of ibuprofen using spray-drying technique. Int. J. Pharm..

[26] Lamprecht A., Saumet J.L., Roux J., Benoit J.P. (2004). Lipid nanocarriers as drug delivery system for ibuprofen in pain treatment. Int. J. Pharm..

[27] Momoh M., Kenechukwu F., Gwarzo M., Builders P. (2015). Formulation and evaluation of ibuprofen loaded lipospheres for effective oral drug delivery. Dhaka Univ. J. Pharm. Sci..

[28] Balleño J.A., Mendizábal-Ruiz A.P., Saade H., Díaz de León-Gómez R., Mendizábal E., Rios-Donato N., López R.G. (2018). Ibuprofen Release from Poly(ethyl cyanoacrylate) Nanoparticles Prepared by Semicontinuous Heterophase Polymerization. Int. J. Polym. Sci..

[29] Marques A.C., Rocha A.I., Leal P., Estanqueiro M., Lobo J.M.S. (2017). Development and characterization of mucoadhesive buccal gels containing lipid nanoparticles of ibuprofen. Int. J. Pharm..

[30] Ćirić A., Milinković Budinčić J., Medarević Đ., Dobričić V., Rmandić M., Barudžija T., Malenović A., Petrović L., Đekić L. (2022). Influence of spray-drying process on properties of chitosan/xanthan gum polyelectrolyte complexes as carriers for oral delivery of ibuprofen. Arh. Za Farm..

[31] Sogias I.A., Williams A.C., Khutoryanskiy V.V. (2012). Chitosan-based mucoadhesive tablets for oral delivery of ibuprofen. Int. J. Pharm..

[32] Shi Y., Xue J., Xu S., You Y., Yan X.Q., Zhao X., Cao J. (2018). Polyelectrolyte complex nanoparticles based on chitosan and methoxy poly(ethylene glycol) methacrylate-co-poly(methylacrylic acid) for oral delivery of ibuprofen. Colloids Surf. B.

[33] Dian L., Yang Z., Li F., Wang Z., Pan X., Peng X., Huang X., Guo Z., Quan G., Shi X. (2013). Cubic phase nanoparticles for sustained release of ibuprofen: Formulation, characterization, and enhanced bioavailability study. Int. J. Nanomed..

[34] John J.E., Tytler B.A., Habila J., Apeji Y.E., Olayemi O., Isimi C.Y. (2022). Cross-linking with multifunctional excipients and its effect on the physicochemical properties and release profile of ibuprofen-loaded Digitaria exilis starch nanoparticles. J. Res. Pham..

[35] Ofokansi K.C., Kenechukwu F.C., Isah A.B., Okigbo E.L. (2013). Formulation and evaluation of glutaraldehyde-crosslinked chitosan microparticles for the delivery of ibuprofen. Trop. J. Pharm. Res..

[36] Anaya Castro M.A., Alric I., Brouillet F., Peydecastaing J., Fullana S.G., Durrieu V. (2018). Soy Protein Microparticles for Enhanced Oral Ibuprofen Delivery: Preparation, Characterization, and In Vitro Release Evaluation. AAPS PharmSciTech.

[37] Mills S.B., Bloch M., Bruckner F. (1973). Double-blind cross-over study of ketoprofen and ibuprofen in management of rheumatoid arthritis. Br. Med. J..

[38] Gleeson S., Sorbie J. (1983). Efficacy of ketoprofen in treating primary dysmenorrhea. Can. Med. Assoc. J..

[39] Pereira-Leite C., Nunes C., Jamal S.K., Cuccovia I.M., Reis S. (2017). Nonsteroidal Anti-Inflammatory Therapy: A Journey Toward Safety. Med. Res. Rev..

[40] Kuczyńska J., Nieradko-Iwanicka B. (2021). Future prospects of ketoprofen in improving the safety of the gastric mucosa. Biomed. Pharmacother..

[41] Cerciello A., Auriemma G., Morello S., Pinto A., Del Gaudio P., Russo P., Aquino R.P. (2015). Design and in Vivo Anti-Inflammatory Effect of Ketoprofen Delayed Delivery Systems. J. Pharm. Sci..

[42] Freitas E.D.d., Lima B.M., Rosa P.C.P., da Silva M.G.C., Vieira M.G.A. (2019). Evaluation of proanthocyanidin-crosslinked sericin/alginate blend for ketoprofen extended release. Adv. Powder Technol..

[43] Franco P., Reverchon E., De Marco I. (2018). PVP/ketoprofen coprecipitation using supercritical antisolvent process. Powder Technol..

[44] Gue E., Willart J.F., Muschert S., Danede F., Delcourt E., Descamps M., Siepmann J. (2013). Accelerated ketoprofen release from polymeric matrices: Importance of the homogeneity/heterogeneity of excipient distribution. Int. J. Pharm..

[45] Browne E., Charifou R., Worku Z.A., Babu R.P., Healy A.M. (2019). Amorphous solid dispersions of ketoprofen and poly-vinyl polymers prepared via electrospraying and spray drying: A comparison of particle characteristics and performance. Int. J. Pharm..

[46] Attia M.F., Anton N., Khan I.U., Serra C.A., Messaddeq N., Jakhmola A., Vecchione R., Vandamme T. (2016). One-step synthesis of iron oxide polypyrrole nanoparticles encapsulating ketoprofen as model of hydrophobic drug. Int. J. Pharm..

[47] Cirri M., Bragagni M., Mennini N., Mura P. (2012). Development of a new delivery system consisting in “drug–in cyclodextrin–in nanostructured lipid carriers” for ketoprofen topical delivery. Eur. J. Pharm. Biopharm..

[48] Savardekar R.Y., Sherikar A.S. (2020). Screening of Ketoprofen-Poloxamer and Ketoprofen-Eudragit solid dispersions for improved physicochemical characteristics and dissolution profile. Braz. J. Pharm. Sci..

[49] Yadav P.S., Kumar V., Singh U.P., Bhat H.R., Mazumder B. (2013). Physicochemical characterization and in vitro dissolution studies of solid dispersions of ketoprofen with PVP K30 and d-mannitol. Saudi Pharm. J..

[50] Cerciello A., Auriemma G., Del Gaudio P., Sansone F., Aquino R.P., Russo P. (2016). A novel core–shell chronotherapeutic system for the oral administration of ketoprofen. J. Drug Del. Sci. Tech..

[51] Das S.K., Khanam J., Nanda A. (2016). Optimization of preparation method for ketoprofen-loaded microspheres consisting polymeric blends using simplex lattice mixture design. Mat. Sci. Eng. C.

[52] Freitas E.D.d., Rosa P.C.P., Silva M.G.C.d., Vieira M.G.A. (2018). Development of sericin/alginate beads of ketoprofen using experimental design: Formulation and in vitro dissolution evaluation. Powder Technol..

[53] Kawata M., Nakamura M., Goto S., Aoyama T. (1986). Preparation and dissolution pattern of Eudragit RS microcapsules containing ketoprofen. Chem. Pharm. Bull..

[54] Lins L.C., Padoin N., Pires A.T.N., Soares C. (2016). Modeling ketoprofen release from PHB/chitosan composite microparticles. Polym. Bull..

[55] Pandit S.S., Hase D.P., Bankar M.M., Patil A.T., Gaikwad N.J. (2009). Ketoprofen-loaded Eudragit RSPO microspheres: An influence of sodium carbonate on in vitro drug release and surface topology. J. Microencapsul..

[56] Yu C., Zhang X., Sun X., Long C., Sun F., Liu J., Li X., Lee R.J., Liu N., Li Y. (2018). Ketoprofen and MicroRNA-124 Co-loaded poly (lactic-co-glycolic acid) microspheres inhibit progression of Adjuvant-induced arthritis in rats. Int. J. Pharm..

[57] Ding S., Serra C.A., Anton N., Yu W., Vandamme T.F. (2019). Production of dry-state ketoprofen-encapsulated PMMA NPs by coupling micromixer-assisted nanoprecipitation and spray drying. Int. J. Pharm..

[58] Aliyah, Saputri E.T., Fitriah D., Arjuna A. (2021). Dissolution improvement of ketoprofen through polymer matrix composite povidone K-30/TiO_2_. J. Exp. Biol. Agric. Sci..

[59] Deák Á., Sebők D., Csapó E., Bérczi A., Dékány I., Zimányi L., Janovák L. (2019). Evaluation of pH-responsive poly(styrene-co-maleic acid) copolymer nanoparticles for the encapsulation and pH- dependent release of ketoprofen and tocopherol model drugs. Eur. Polym. J..

[60] Boppana R., Yadaorao Raut S., Krishna Mohan G., Sa B., Mutalik S., Reddy K.R., Das K.K., Biradar M.S., Kulkarni R.V. (2019). Novel pH-sensitive interpenetrated network polyspheres of polyacrylamide-g-locust bean gum and sodium alginate for intestinal targeting of ketoprofen: In vitro and in vivo evaluation. Colloids Surf. B.

[61] Widyastama G., Kurniati M. (2021). Optimization of Sonication Time and Surfactant Concentration for Chitosan-alginate Coated Ketoprofen Nanoencapsulation. Journal of Physics: Conference Series.

[62] Stigliani M., Aquino R.P., Del Gaudio P., Mencherini T., Sansone F., Russo P. (2013). Non-steroidal anti-inflammatory drug for pulmonary administration: Design and investigation of ketoprofen lysinate fine dry powders. Int. J. Pharm..

[63] Talele S., Nikam P., Ghosh B., Deore C., Jaybhave A., Jadhav A. (2017). A research article on nanogel as topical promising drug delivery for diclofenac sodium. Indian J. Pharm. Educ. Res..

[64] Ghalei S., Asadi H., Ghalei B. (2018). Zein nanoparticle-embedded electrospun PVA nanofibers as wound dressing for topical delivery of anti-inflammatory diclofenac. J. Appl. Polym. Sci..

[65] Nguyen C.N., Nguyen T.T.T., Nguyen H.T., Tran T.H. (2017). Nanostructured lipid carriers to enhance transdermal delivery and efficacy of diclofenac. Drug Deliv. Transl. Res..

[66] Küçüktürkmen B., Umut Can Ö., Bozkir A. (2017). In situ hydrogel formulation for intra-articular application of diclofenac sodium-loaded polymeric nanoparticles. Turk. J. Pharm. Sci..

[67] Butoescu N., Jordan O., Doelker E. (2009). Intra-articular drug delivery systems for the treatment of rheumatic diseases: A review of the factors influencing their performance. Eur. J. Pharm. Biopharm..

[68] Rattes A.L.R., Oliveira W.P. (2007). Spray drying conditions and encapsulating composition effects on formation and properties of sodium diclofenac microparticles. Powder Technol..

[69] Ahmed M.M., El-Rasoul S.A., Auda S.H., Ibrahim M.A. (2013). Emulsification/internal gelation as a method for preparation of diclofenac sodium–sodium alginate microparticles. Saudi Pharm. J..

[70] Desai K.G. (2007). Properties of tableted high-amylose corn starch-pectin blend microparticles intended for controlled delivery of diclofenac sodium. J. Biomater. Appl..

[71] Remunan-Lopez C., Lorenzo-Lamosa M., Vila-Jato J., Alonso M. (1998). Development of new chitosan–cellulose multicore microparticles for controlled drug delivery. Eur. J. Pharm. Biopharm..

[72] Franco P., Reverchon E., De Marco I. (2018). Zein/diclofenac sodium coprecipitation at micrometric and nanometric range by supercritical antisolvent processing. J. CO2 Util..

[73] Musumeci T., Bonaccorso A., Carbone C., Impallomeni G., Ballistreri A., Duskey J.T., Puglisi G., Pignatello R. (2020). Development and biocompatibility assessments of poly(3-hydroxybutyrate-co-ε-caprolactone) microparticles for diclofenac sodium delivery. J. Drug Del. Sci. Tech..

[74] Ray S., Banerjee S., Maiti S., Laha B., Barik S., Sa B., Bhattacharyya U.K. (2010). Novel interpenetrating network microspheres of xanthan gum–poly (vinyl alcohol) for the delivery of diclofenac sodium to the intestine—In vitro and in vivo evaluation. Drug Deliv..

[75] Deshmukh R., Naik J. (2014). Study of formulation variables influencing polymeric microparticles by experimental design. ADMET DMPK.

[76] Barzegar-Jalali M., Alaei-Beirami M., Javadzadeh Y., Mohammadi G., Hamidi A., Andalib S., Adibkia K. (2012). Comparison of physicochemical characteristics and drug release of diclofenac sodium–eudragit^®^ RS100 nanoparticles and solid dispersions. Powder Technol..

[77] Duarte Junior A.P., Tavares E.J.M., Alves T.V.G., de Moura M.R., da Costa C.E.F., Silva Júnior J.O.C., Ribeiro Costa R.M. (2017). Chitosan nanoparticles as a modified diclofenac drug release system. J. Nanoparticle Res..

[78] Li X., Zhang Z., Li J., Sun S., Weng Y., Chen H. (2012). Diclofenac/biodegradable polymer micelles for ocular applications. Nanoscale.

[79] Sandeep C., Deb T., Moin A., Hg S. (2014). Cationic guar gum polyelectrolyte complex micro particles. J. Young Pharm..

[80] Curcio M., Gianfranco Spizzirri U., Iemma F., Puoci F., Cirillo G., Parisi O.I., Picci N. (2010). Grafted thermo-responsive gelatin microspheres as delivery systems in triggered drug release. Eur. J. Pharm. Biopharm..

[81] Tawfeek H.M. (2013). Evaluation of PEG and mPEG-co-(PGA-co-PDL) microparticles loaded with sodium diclofenac. Saudi Pharm. J..

[82] Murtaza G., Ahmad M., Shahnaz G. (2010). Microencapsulation of diclofenac sodium by nonsolvent addition technique. Trop. J. Pharm. Res..

[83] Liu C.-S., Desai K.G.H., Meng X.-H., Chen X.-G. (2007). Sweet Potato Starch Microparticles as Controlled Drug Release Carriers: Preparation and In Vitro Drug Release. Dry. Technol..

[84] Narayanan D., Pillai G.J., Nair S.V., Menon D. (2019). Effect of formulation parameters on pharmacokinetics, pharmacodynamics, and safety of diclofenac nanomedicine. Drug Deliv. Transl. Res..

[85] Wen X., Huang X., Wu H. (2021). Development of a novel intraarticular injection of diclofenac for the treatment of arthritis: A preclinical study in the rabbit model. Acta Biochim. Pol..

[86] Badaoui F.Z., Bouzid D. (2022). Formulation and Optimization of Diclofenac Sodium Loaded Ethylcellulose Nanoparticles. Braz. J. Pharm. Sci..

[87] Cetin M., Atila A., Kadioglu Y. (2010). Formulation and in vitro characterization of Eudragit^®^ L100 and Eudragit^®^ L100-PLGA nanoparticles containing diclofenac sodium. AAPS PharmSciTech.

[88] Gavini E., Spada G., Rassu G., Cerri G., Brundu A., Cossu M., Sorrenti M., Giunchedi P. (2011). Development of solid nanoparticles based on hydroxypropyl-β-cyclodextrin aimed for the colonic transmucosal delivery of diclofenac sodium. J. Pharm. Pharmacol..

[89] Meneguin A.B., da Silva Barud H., Sábio R.M., de Sousa P.Z., Manieri K.F., de Freitas L.A.P., Pacheco G., Alonso J.D., Chorilli M. (2020). Spray-dried bacterial cellulose nanofibers: A new generation of pharmaceutical excipient intended for intestinal drug delivery. Carbohydr. Polym..

